# Insights into pituitary stalk interruption syndrome: 2 cases that illuminate the condition

**DOI:** 10.1016/j.radcr.2024.11.065

**Published:** 2024-12-19

**Authors:** Fadwa Jaheddine, Nazik Allali, Siham El Haddad, Latifa Chat

**Affiliations:** Pediatric Radiology Department, Children's Hospital, University Mohammed V of Rabat, Rabat, Morocco

**Keywords:** Pituitary stalk interruption syndrome, PSIS, MRI, Growth deficiency, Short stature

## Abstract

Pituitary stalk interruption syndrome (PSIS) is a congenital anatomical defect that leads to pituitary insufficiency, The symptoms are diverse, often leading to diagnostic delays or even misdiagnosis. MRI plays a crucial role in establishing an accurate diagnosis by revealing a characteristic radiological triad: a thin or absent pituitary stalk, an ectopic or missing posterior pituitary gland, and anterior pituitary hypoplasia. We herein describe 2 cases: 1 involving a 9-year-old boy and the other an 11-year-old girl, both diagnosed with PSIS.

## Introduction

Pituitary Stalk Interruption Syndrome (PSIS) is an exceptionally rare condition that can lead to growth failure and delayed onset of puberty. The precise cause of PSIS remains unclear. Diagnosis typically involves the use of magnetic resonance imaging (MRI) to examine the hypothalamus and pituitary gland, which may reveal features such as an ectopic or absent posterior pituitary, a disrupted or absent pituitary stalk, or a small anterior pituitary gland, alongside deficiencies in growth hormone or other pituitary hormones [1].

We present 2 cases of children admitted with GH deficiency, diagnosed with PSIS, confirmed by pituitary MRI.

## Case presentation 1

A 9-year-old boy was admitted to the pediatric department for evaluation of short stature. He had no significant medical or family history. Physical examination confirmed short stature; he weighed 30 kg and measured 107.5 cm in height, which placed him at 3 standard deviations (SD) below the mean compared to his mid-parental predicted height. Testicular volume, pubic hair development, and penile size were all consistent with Tanner stage 1.

The laboratory results indicated a low level of IGF1 at 40ng/mL (Normal range: 120–900ng/mL).

Bone age was 7 years according to the Greuliche and Pyle method.

**Fig. 1:**
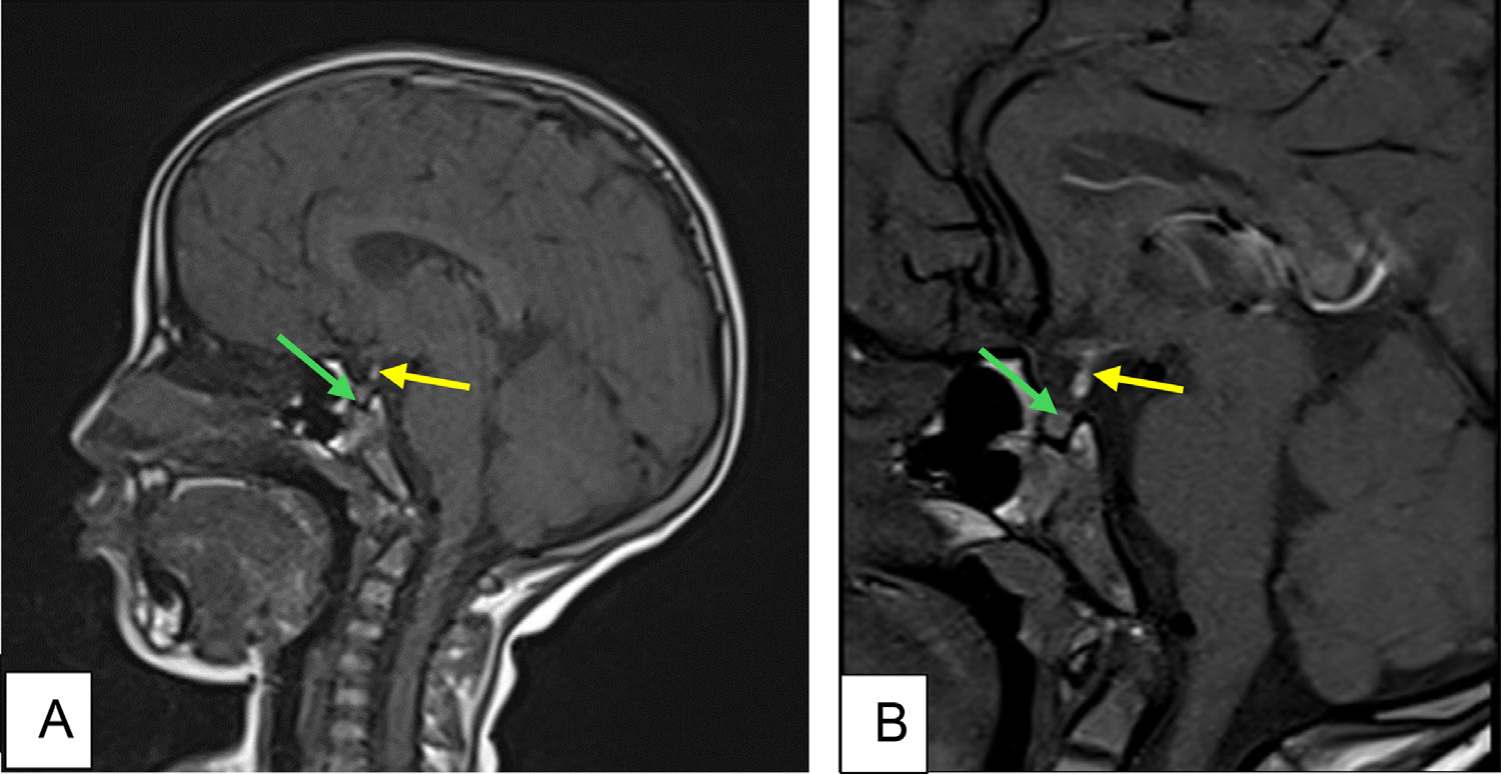
Pituitary MRI finding. (A) And (B) Sagittal Pre contrast and Post contrast T1 weighted images show a hypoplastic anterior pituitary gland (green arrow) measuring 2.5 mm (Normal: 4.5±0.6mm) with an ectopic posterior pituitary located at the distal end of the pituitary stalk (yellow arrow). Note the absence of the identifiable pituitary stalk.

Pituitary MRI revealed a hypoplastic anterior pituitary gland, with an absent pituitary stalk and an ectopic posterior pituitary located at the distal end of the pituitary stalk (Fig. 1). There was no associated abnormality of the central nervous system.

The MRI finding were consistent with PSIS.

The patient was placed on recombinant human growth hormone (rhGH) replacement therapy. The progression was characterized by a height gain of 5 cm in the first year, reaching a total height of 112.5 cm.

## Case presentation 2

A previously healthy 11-year-old girl was referred to the pediatric department for evaluation of short stature, first noticed by her parents in comparison to her age-matched peers. There was no relevant medical or family history of growth disorders or endocrine issues. On clinical examination, her height was recorded at 123 cm and her weight at 40 kg, corresponding to 3.5 standard deviations below the mean for her age and significantly below her mid-parental target height. There was no evidence of pubertal development, as breast development and pubic hair were absent, and she had not yet experienced menarche, indicating a prepubertal status consistent with Tanner Stage 1.

The laboratory results indicated a low level of IGF1 at 50ng/mL (Normal range: 120–900ng/mL).

Bone age was 8 years according to the Greuliche and Pyle method.

**Fig. 2:**
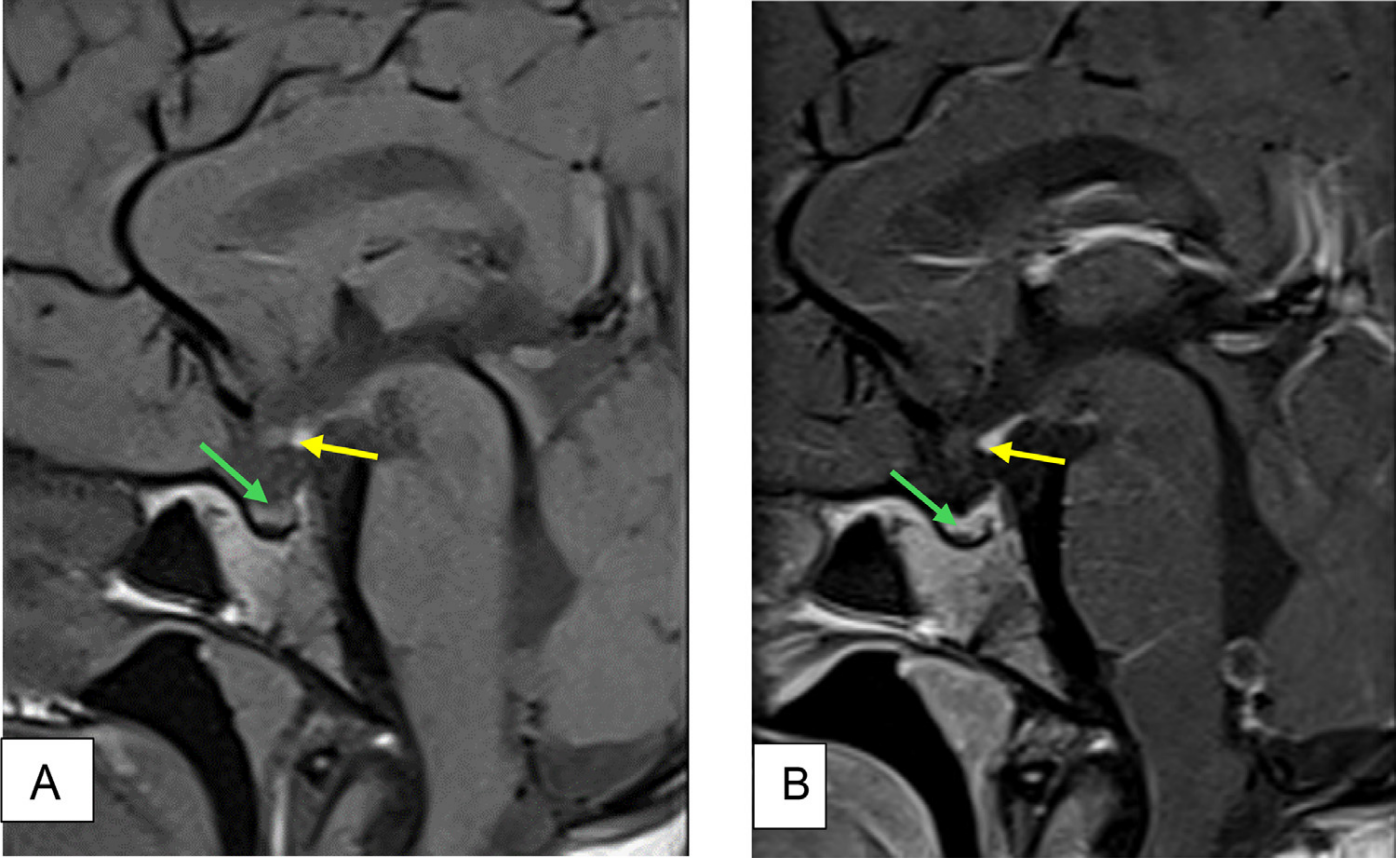
(A) And (B) Sagittal Pre contrast and Post contrast T1 weighted images show hypoplastic anterior pituitary gland (green arrow) measuring 2 mm (Normal: 5±2 mm) with an ectopic posterior pituitary (yellow arrow). Note the absence of the identifiable pituitary stalk.

Pituitary MRI was performed, it revealed a hypoplastic anterior pituitary gland, with an absent pituitary stalk and an ectopic posterior pituitary (Fig. 2). There was no associated abnormality of the central nervous system.

The MRI finding were consistent with PSIS.

The patient was placed on recombinant human growth hormone (rhGH) replacement therapy.The progression was characterized by a height gain of 4.5 cm in the first year, reaching a total height of 127.5 cm.

## Discussion

Pituitary stalk interruption syndrome (PSIS) was first identified and characterized in 1987 by Fujisawa et al. [2]. Its epidemiology shows an incidence of approximately 0.5 per 100,000 live births [3]. The average age at diagnosis ranges from 9.4 to 11.6 years [4].

The exact cause of PSIS remains uncertain, though various hypotheses have been proposed. These include perinatal trauma and abnormal organ development linked to genetic or environmental factors during fetal development. Rare mutations in genes such as HESX1, LH4, OTX3, and SOX3 have been identified, particularly in familial PSIS cases. Additionally, 20%-50% of PSIS patients present with other congenital abnormalities, often involving midline defects like cleft lip, diaphragmatic agenesis, optic nerve hypoplasia, encephalocele, or harelip. This suggests that the implicated genetic mutations may disrupt the embryonic development of the hypothalamic-pituitary axis [5].

Due to variations in age and the extent of pituitary stalk damage, the clinical presentation of the PSIS can be complex and diverse. In neonates, common symptoms include hypoglycemia, prolonged jaundice, micropenis, and cryptorchidism. In older children and adults, the syndrome primarily manifests as growth retardation, short stature, and abnormalities in sexual development. Studies indicate that all PSIS patients have growth hormone deficiency, while 97.2% show gonadotropin deficiency, 88.2% have adrenocorticotropic hormone (ACTH) deficiency, and 70.3% experience thyroid-stimulating hormone (TSH) deficiency [6].

MRI is the imaging modality of choice for evaluating the hypothalamic-pituitary region in infants and young children. Standard protocols include high-resolution T1- and T2-weighted images with 2-mm to 3-mm slice thickness in both coronal and sagittal planes. Sagittal T1 images are ideal for assessing the size and contour of the anterior pituitary and for visualizing the size and position of the posterior pituitary bright spot. Coronal views provide excellent detail of the anterior pituitary, pituitary stalk, optic chiasm, and parasellar regions. Dynamic contrast-enhanced imaging offers additional insight into the vascular aspects of the pituitary stalk. Axial T2-weighted imaging of the entire brain is recommended to detect other potential abnormalities [7].

Pituitary MRI is the best diagnostic tool for the diagnosis of PSIS. It shows a characteristic radiological triad: absence of the pituitary stalk, anterior pituitary hypoplasia (98.3%), and posterior pituitary ectopia (91.2%). Neurohypophyseal ectopia is frequently found in the infundibular remnant (60.4%) [8].

Currently, Pituitary Stalk Interruption Syndrome cannot be cured through medication or surgical interventions. The treatment consists of providing physiological doses of hormone replacement therapy, underscoring the significance of early detection of hormone deficiencies for effective management [6].

## Conclusion

Early diagnosis and management of PSIS via hormone replacement therapy are essential for preventing long-term complications. Since the diagnosis primarily relies on MRI finding, radiologists should be aware of this rare cause of GH deficiency.

## Patient consent

Written informed consent was obtained from the legal authorized representative of the patient for the publication of this case report.

## References

[1] Alali I, Saad R, Kabalin Y (2020). Two cases of pituitary stalk interruption syndrome in Syrian children. Case Rep Endocrinol.

[2] Yehouenou Tessi RT, Adeyemi B, El Msaadi S, El Haddad S, Allali N, Chat L (2023). Pituitary stalk interruption syndrome on MRI: case report. Clinical Case Reports.

[3] Fatima T, Hussain Chandio S, Muzaffar K, Mumtaz H, Jahan N (2020). Pituitary stalk interruption syndrome. Cureus.

[4] Alqarni AA, Abdalla KM, Alqarni MAS, Alfaifi JA, Osman HG, Al Alhindi BS (2024). Pituitary stalk interruption syndrome: a case report and literature review. Ann Med Surg.

[5] Dawadi K, Dahal P, Poudyal B (2023). Pituitary stalk interruption syndrome: a case report. Radiol Case Rep.

[6] Wu R, Xu J (2024). Pituitary stalk interruption syndrome with excessive height growth combined with congenital absence of the uterus and ovaries: a rare case report and review of the literature. Diabetes Metab Syndr Obes.

[7] Parks JS (2018). Congenital hypopituitarism. Clin Perinatol.

[8] Wang Q, Hu Y, Li G, Sun X (2014). Pituitary stalk interruption syndrome in 59 children: the value of MRI in assessment of pituitary functions. Eur J Pediatr.

